# Social and physical factors related to depression in the older population of Siberia, Russia: a cross-sectional study

**DOI:** 10.1186/s12877-021-02225-7

**Published:** 2021-04-23

**Authors:** Georg von Fingerhut, Katsuyoshi Mizukami, Dorothy Yam, Konstantin Makarov, Yuriy Kim, Elena Kondyurina, Lyudmila Yakovleva

**Affiliations:** 1 Department of Gerontological Nursing and Caring, Human Care Science, Graduate School of Comprehensive Human Sciences, University of Tsukuba, Comprehensive Research Building D-310, 1-1-1 Tennodai, Tsukuba, Ibaraki, 305-8577 Japan; 2 Faculty of Health and Sport Sciences, Graduate School of Comprehensive Human Sciences, University of Tsukuba, Comprehensive Research Building D-311, 1-1-1 Tennodai, Tsukuba, Ibaraki, 305-8577 Japan; 3 Department of Neuroscience, Brown University, 185 Meeting St, Providence, RI 02912 USA; 4 Department of Obstetrics and Gynecology, Novosibirsk State Medical University, 52, Krasny Prospect, Novosibirsk, Russian Federation 630091; 5 Department of Nursing, Novosibirsk State Medical University, 52, Krasny Prospect, Novosibirsk, Russian Federation 630091; 6 Department of Pediatrics, Novosibirsk State Medical University, 52, Krasny Prospect, Novosibirsk, Russian Federation 630091

**Keywords:** Older individuals, Depression, Geriatric depression scale, Cold climate, Russia

## Abstract

**Background:**

Depression and suicide rates are relatively high in the colder regions of Russia. Older individuals in these regions are especially susceptible to these issues and are understudied in this regard. This study aims to better understand the current depression prevalence, and the factors related to depression, among the older individuals in these colder regions of Russia by studying a population in Novosibirsk oblast.

**Methods:**

A questionnaire survey was administered to 422 older individuals, assessing basic attributes and health status, and employing the following standardized scales: 8-item Short-Form Health Survey, Pittsburgh Sleep Quality Index, and 15-item Geriatric Depression Scale (GDS). Participants were divided in two groups (GDS ≤ 6, GDS > 6) and compared, using Student’s t test, χ2 test, and logistic regression analysis.

**Results:**

Young old (YO) adults showed significant correlation of depression with asthma (*P = 0.005, OR = 6.40,* 95%CI: *1.74–23.5*), having a spouse (*P = 0.016, OR = 1.99,* 95%CI: *1.14–3.4*8), and daily communication with others (*P < 0.001, OR = 0.336,* 95%CI: *0.197–0.572*). Among old old (OO) adults, significant correlation with depression was found for the variables work status (*P = 0.047, OR = 0.115,* 95%CI: *0.014–0.974*), and weekly walking (*P = 0.014, OR = 0.288,* 95%CI: *0.106–0.778*).

**Conclusions:**

Twenty eight percent of the participants have depression. In YO adults, frequent communication and social ties with individuals outside of the family can mitigate depression prevalence. As for OO adults, the factors that have the highest impact on mitigating depression are related to daily activity, including both frequent walking and working or self-employment. Asthma patients are one of the more sensitive groups towards depression, but further research on this topic is needed.

**Supplementary Information:**

The online version contains supplementary material available at 10.1186/s12877-021-02225-7.

## Introduction

In 2016, Russia was the country with the second highest mortality by suicide rate, with 31 suicides recorded per 100,000 people [1]. One of the leading factors contributing to suicide in Russia is depression, such that 90% of untreated depression cases end in suicide [2]. The region with the highest suicide and mortality rates is the Siberian federal district, with both 50.7 cases of suicide and 96.6 cases of depression reported per 100,000 people [3]. The Siberian federal district happens to be one of the coldest in Russia [4]. Previous research has shown that human habitation in the colder regions of Russia is accompanied by a restructuring of regulatory mechanisms of the neuroendocrine system, particularly hypothalamic–pituitary–thyroid axis, responsible for stress response and metabolism [5]. Climatic and geographic factors can have aggrieving effects on human health within these colder regions of Russia, causing young people to form psychoemotional stress due to higher secretion of the hormone cortisol into the blood [6]. Depressed patients in the colder regions of Russia also show lower scores in scales for both physical and mental components of quality of life [7]. As individuals age, there is higher chance of developing other illnesses which can be comorbid with depression, such as asthma [8] (which is especially affected by the cold climate [9]). Besides environmental and biological factors, social factors such as gender also undoubtedly play a large role in depression. Moreover, marital status, living conditions, and other social factors, highly influence depression prevalence and greatly vary with age [10]. As for differences within older age groups, depression prevalence in young old (YO; ages 60 to 75) and old old (OO; ages 76 to 93) adults varies slightly, and is much harder to differentiate due to confounding variables such as illness and biochemical changes in the aging brain [10]. As for the Russian Federation, one of the first studies focusing on the differences in depression prevalence separately in YO and OO adults appeared only in 2020 [11], as Podkovina et al. studied 380 older patients in Smolensk region and pointed out that depression prevailed in one third of YO patients, whereas in the OO it was almost 50% [11]. Moreover, the authors drew attention to the association of depression with senile asthenia, one of the most common diseases of older age, especially in OO patients [11]. These facts point out the importance of understanding the causes for depression in older age and underscore the importance of separating YO and OO adults in research. The low life expectancy of Russian population and the low population density of the colder regions make it difficult to conduct the research among older Russian individuals, and particularly difficult to assess the difference between YO and OO adults in the colder regions. Therefore the current depression prevalence among community-dwelling older individuals, and the specific social factors linked to depression among the Russian elders living in the colder regions of Russia, are still widely unknown.

The present study takes place in Novosibirsk oblast, within the Siberian federal district. An oblast is a type of federal subject of the Russian Federation. The Siberian federal district makes up 25.5% of Russian territory, with 4,361,757 km^2^ of land, and is the third largest district in Russia by population with 17,173,335 people (11.7%) [4]. The main oblast of Siberian federal district is Novosibirsk oblast, in which 14.2% of the population is 65 and older, and the average life expectancy as 71.9 years, which is almost the same as the average value in Russia [3]. The capital of Novosibirsk oblast is Novosibirsk city, which is located in the northern latitudes (55.1812° N, 82.5523° E). It is both one of the three largest cities in Russia by population and the central city of Siberia, where winter is long and the population density is as low as 3.34 person/km^2^ [4]. The average temperature in the region is 1.8 °C, the minimum temperature is − 51 °C, and there is an average of 67 days without sunshine every year in Novosibirsk oblast [4]. The population of Novosibirsk oblast is the highest in the Siberian federal district and it includes many ethnic representatives of the other oblasts and cities of the district due to high urbanization in the region [12], making it an ideal location from which to gather a sample. In a previous study in Russia, surveying 16,877 participants, it was found that 25.6% of the population has depression [13]. In a study done on Canada, a region of similar climate and latitude, approximately 14% of the population was said to have depression. Not only do the rates in Russia appear higher, but also less is known about the characteristics of depression in older populations of the region.

The purpose of this study is to examine factors related to the current depression prevalence and the relationship between depression and social factors among community-dwelling older individuals living in the Siberian federal district of Russia.

## Material and methods

### Study design and subjects

This cross-sectional study employs observational research methods, using questionnaire surveys to collect data.

The sample was comprised of 422 older individuals, aged 60 years or older, living in Novosibirsk oblast. Recruitment of participants for this study was conducted after contacting representatives from local governmental social centers and geriatric health support organizations for the contacts of older individuals living in the Novosibirsk oblast. Healthy and self-sufficient individuals with ability to answer subjective questionnaires were included in the study. Exclusion criteria included significant illness, cognitive impairment, and alcohol dependence. Due to the sensitivity of cognitive impairment examinations, such as the Mini-Mental State Examination, to ethnicity, age, and educational background [14], we verbally obtained collateral information from family members regarding the decrease of cognitive function in their daily life situations to additionally evaluate cognitive impairment. Written consent was obtained from the subjects before they completed the self-report questionnaires. The ethics committee at the University of Tsukuba Medical System approved this study (N1307) before research was conducted. To maintain anonymity of the participants, an ID number was created for each participant, and the gathered data were analyzed.

The surveys were administered in Novosibirsk oblast from March 4, 2019 to March 29, 2019. The basic attributes collected from participants were age, gender, height, weight, presence or absence of disease (diabetes, asthma, obstructive sleep apnea), family structure and living situation, monthly income, working status (including self-employment), smoking history or current smoking status, current drinking status, frequency of daily communication with others, favorite person with whom to talk (spouse, kids, siblings, relatives, friends), and frequency of walking per week. The 8-item Short-Form Health Survey (SF-8) was used to measure subjective health and assess health-related quality of life, showing both the Physical Component Summary (PCS) and Mental Component Summary (MCS). Furthermore, 15-items Geriatric Depression Scale (GDS) was used to measure subjects’ depression level. The Pittsburgh Sleep Quality Index (PSQI) was used to measure sleep quality.

### Statistical analyses

The Mann–Whitney U test, the Student’s *t* test, the χ^2^ test, and logistic regression analysis were conducted using the Statistical Package for Social Sciences (SPSS), version 24. The Shapiro-Wilks test was used to examine normality. The two-samples Student’s *t* test (unpaired) was used for variables with normality, and the Mann-Whitney U test was used for variables without normality. The χ^2^ test was used for categorical variables. The *P*-values less than 0.05 were considered significant. Backward elimination (likelihood ratio) was used in the logistic regression analysis for all the correlations with *P*-value less than 0.15. The sample was stratified by age for the latter analyses, with YO adults ranging in age from 60 to 75, and OO adults aged 76 to 93.

GDS score was subdivided into two categories, with 5/6 as a cut-off point to screen for depression in Russia [15]. For statistical analysis, the sample was split into two main groups based on depression scores, with a GDS score ≥ 6 indicating the depression group, and GDS score < 6 indicating the non-depression group. Regarding the quality of sleep of the older people, a PSQI score of 5/6 was used as the cutoff point, where a score of 6 and higher is regarded as sleep disorder [16]. The monthly income was also used to divide the sample into two groups, based on the minimum living standard in Novosibirsk oblast (8524 rubles) [3]. Frequency of walking per week was divided by the mean 4.0 (standard deviation (SD): 1.5) times per week, due to the normal distribution. Frequency of communication with others was divided by the median 3.0 (SD: 1.8) times per day due to the non-normal distribution.

## Results

Answers to the questionnaires were obtained from all 422 participants. Participants had a median 3.0 (interquartile range: 1–6) GDS score; 118 individuals (28%) had depression (GDS ≥6), while 304 (72%) did not. There were 331 (78.4%) YO adults and 91 (21.6%) OO adults. Among OO adults, depression prevalence was slightly higher (29.6%, compared to 27.5% of young-old adults), however no significance was found (*p = 0.694*).

The average age of the sample was 71.3 years, average BMI was 27.9, and the gender breakdown was 226 males (53.6%) and 196 females (46.4%). The depression prevalence in female was 28.1%, slightly higher than in males (27.9%), but with no significant difference (*p = 1.00*). The average PCS of SF-8 was 39.9, although no significant difference between the depression and non-depression groups was found. The average MCS of SF-8 for the entire sample was 47.6, which is slightly lower than average Russian population score amongst individuals no self-reported depression (48.8) [17]. Regarding the diseases reported by participants, 54 (12.8%) had diabetes and 19 (4.5%) had asthma. The prevalence of asthma is significantly higher in the depression group (*P* = .019). Regarding sleep quality, 257 (60.9%) of the participants had sleep disorder (PSQI ≥6), whereas 94 (22.3%) had obstructive sleep apnea syndrome. Currently smoking or had smoked in the past were 139 (32.9%), and non-smoking participants showed significantly lower depression scores (*P* = .038) Eighty-six of the participants (28.3%) were working or self-employed, and those who worked showed predominantly lower depression scores (*P* < .001). Within the sample, 234 participants (55.4%) reported having a spouse, and significantly higher number of individuals had a spouse in the depressive group (*P* = .012). As per communication variables, individuals who spoke with others daily 3 times or more included 218 individuals (51.6%), showing significant difference between the depressed and non-depressed group (*P* < .001). Sixty-three of participants reported a friend as their favorite person with whom to talk, and among these individuals, 54 (17.8%) had no depression, while only 9 (7.6%) were depressed (*P* = .009). As per physical activity, 323 (76.5%) reported walking 4 days or more per week, showing significant difference between the non-depressed and depressed groups (*P* = .040) (Table 1).

**Table 1:** Participants’ characteristics

				*N* = 422
	Total N = 422	Normal (GDS < 6), *n* = 304	Depression (GDS ≥ 6), *n* = 118	*p*
Young Old Adults (60–75)	331 (78.4%)	240 (78.9%)	91 (77.1%)	.694
Older Old Adults (76–93)	91 (21.6%)	64 (21.1%)	27 (22.9%)	.694
Gender				
Male	226 (53.6%)	163 (53.6%)	63 (53.4%)	1.00
Female	196 (46.4%)	141 (46.4%)	55 (46.6%)	
BMI (mean ± SD)	27.9 ± 4.9	27.4 ± 5.6	27.2 ± 4.6	.576†
PCS (SF-8) (mean ± SD)	39.9 ± 8.2	35.1 ± 8.7	36.4 ± 9.3	.205†
MCS (SF-8) (mean ± SD)	47.6 ± 6.6	46.7 ± 8.0	46.2 ± 7.2	.533†
Diabetes				
Yes	54 (12.8%)	34 (11.2%)	20 (16.9%)	.143
No	368 (87.2%)	270 (88.8%)	98 (83.1%)	
Asthma				
Yes	19 (4.5%)	9 (3.0%)	10 (8.5%)	.019*
No	403 (95.5%)	295 (97.1%)	108 (91.5%)	
Sleep Disorder (PSQI 5/6)				
Yes	257	185	72	1.00
No	165	119	46	
Obstructive Sleep Apnea				
Yes	94 (22.3%)	74 (24.3%)	20 (16.9%)	.118
No	328 (77.7%)	230 (75.7%)	98 (83.1%)	
Current Drinking Status				
Yes	305 (72.3%)	222 (73.0%)	83 (70.3%)	.628
No	117 (27.7%)	82 (27.0%)	35 (29.7%)	
Currently Smoking or Smoked in the Past				
Yes	139 (32.9%)	91 (29.9%)	48 (40.7%)	.038*
No	283 (67.1%)	213 (70.1%)	70 (59.3%)	
Currently Working or Self-employed				
Yes	99 (23.5%)	86 (28.3%)	13 (11.0%)	.000**
No	323 (76.5%)	218 (71.7%)	105 (89.0%)	
Monthly Income (ruble)				
≤ 8524	60	45	15	.644
> 8524	362	259	103	
Kid(s)				
Yes	102 (24.2%)	67 (22.0%)	35 (29.7%)	.128
No	320 (75.8%)	237 (78.0%)	83 (70.3%)	
Spouse				
Yes	234 (55.4%)	157 (51.6%)	77 (65.2%)	.012*
No	188 (44.6%)	147 (48.4%)	41 (34.3%)	
Living Alone				
Yes	121 (28.7%)	94 (30.9%)	27 (22.9%)	.119
No	301 (71.3%)	210 (69.1%)	91 (77.1%)	
Daily Communication with Others				
> 3 times	218 (51.6%)	179 (58.9%)	39 (33.0%)	.000**
≤ 2 times	204 (48.4%)	125 (41.1%)	79 (67.0%)	
Favorite Person for Talk: Spouse				
Yes	145 (34.4%)	104 (34.2%)	41 (34.8%)	.910
No	277 (65.6%)	200 (65.8%)	77 (65.2%)	
Favorite Person for Talk: Kids				
Yes	114 (27.0%)	79 (26.0%)	35 (29.7%)	.465
No	308 (73.0%)	225 (74.0%)	83 (70.3%)	
Favorite Person for Talk: Siblings				
Yes	16 (3.8%)	14 (4.6%)	2 (1.7%)	.255
No	406 (96.2%)	290 (95.3%)	116 (98.3%)	
Favorite Person for Talk: Relatives				
Yes	52 (12.3%)	42 (13.8%)	10 (8.5%)	.186
No	370 (87.7%)	262 (86.2%)	108 (91.5%)	
Favorite Person for Talk: Friends				
Yes	63 (14.9%)	54 (17.8%)	9 (7.6%)	.009*
No	359 (85.1%)	259 (85.2%)	109 (92.4%)	
Weekly Walking				
≥ 4 days	323 (76.5%)	241 (79.3%)	82 (69.5%)	.040*
< 4 days	99 (23.5%)	63 (20.7%)	36 (30.5%)	

*Not*e: *p < 0.05, **p < 0.01 χ^2^ test (unpaired), †: Student’s T-test mean ± SD. *BMI* Body Mass Index, *PCS* Physical Component Summery of Short Form-8, *MCS* Mental Component Summery of Short Form-8, *GDS* Geriatric Depression Scale, *PSQI* Pittsburgh Sleep Quality Index

The logistic regression analysis and backward elimination results showed that depression among older Russian people was significantly associated with having asthma (*P* = 0.031, *OR = 2.95, 95%CI: 1.10–7.92*), work or self-employment status (*P* = 0.010, *OR = 0.421, 95%CI: 0.218–0.810*), having a spouse (*P* = 0.036, *OR = 1.65, 95%CI: 1.03–2.64*) and daily communication with others (*P* < 0.001, *OR = 0.346, 95%CI: 0.217–0.551*) (Table 2).

**Table 2:** Logistic regression analysis of depression prevalence among community-dwelling older people living in colder regions of Russia

			N = 422
	Total		
Factor	Odds Ratio	95% CI: LL - UL	*P*-value
Asthma	2.95	1.10–7.92	.031*
Obstructive Sleep Apnea	.609	.336–1.10	.102
Currently working or self-employed	.421	.218–.810	.010*
Kids	1.63	.979–2.73	.060
Spouse	1.65	1.03–2.64	.036*
Daily Communication to Others	.346	.217–.551	>.001**
Weekly Walking	–	–	–

*Not*e: Backward Elimination (Likelihood Ratio): Diabetes (No = 0, Yes = 1), Asthma (No = 0, Yes = 1), Obstructive Sleep Apnea (No = 0, Yes = 1), Current Smoking or Smoked in the Past Status (No = 0, Yes = 1), Currently Working or Self-employed (No = 0, Yes = 1), Kids (No = 0, Yes = 1), Spouse (No = 0, Yes = 1), Living Alone (No = 0, Yes = 1), Daily Communication to Others (≤2 times =0, > 3 times = 1), Favorite Person to Talk: Friends (No = 0, Yes = 1), Weekly Walking (< 4 days = 0, ≥4 days = 1). Hosmer-Lemeshow test *p* = .642 (Total), *p* = .454 (YO), *p* = .986 (OO), the discriminant predictive value = 71.3% (Total), 63.7% (YO), 70.1% (OO), cut-off point: *GDS* Geriatric Depression Scale < 6 = 0, GDS ≥ 6 = 1, *p < 0.05, **p < 0.01. *CI* Confidence interval, *LL* Lower limit, *UL* Upper limit

After stratification by age group, YO adults (age 60 to 75) showed significant correlation of depression with asthma (*P* = 0.005, *OR = 6.40, 95%CI: 1.74–23.5*), having a spouse (*P* = 0.016, *OR = 1.99, 95%CI: 1.14–3.48*) and daily communication with others (*P* < 0.001, *OR = 0.336, 95%CI: 0.197–0.572*). Among OO adults, significant correlation with depression was found for the variables work status (*P* = 0.047, *OR = 0.115, 95%CI: 0.014–0.974*) and weekly walking (*P* = 0.014, *OR = 0.288, 95%CI: 0.106–0.778*) (Table 3).

**Table 3:** Stratified analysis by age group of depression prevalence among community-dwelling older people living in colder regions of Russia

						N = 422
	YO, *n* = 331			OO, *n* = 91		
Factor	Odds Ratio	95% CI: LL - UL	*P*-value	Odds Ratio	95% CI: LL - UL	*P*-value
Asthma	6.40	1.74–23.5	.005*	–	–	–
Obstructive Sleep Apnea	–	–	–	–	–	–
Currently working or self-employed	.511	.253–1.03	.061	.115	.014–.974	.047*
Kids	1.78	.979–3.23	.059	–	–	–
Spouse	1.99	1.14–3.48	.016*	–	–	–
Daily Communication to Others	.336	.197–.572	>.001**	.410	.142–1.19	.100
Weekly Walking	–	–	–	.288	.106–.778	.014*

*Not*e: Stratified analysis by age group, *YO* Young old adults 60–75 years old, *OO* Older old adults 76–93 years old. Backward Elimination (Likelihood Ratio): Diabetes (No = 0, Yes = 1), Asthma (No = 0, Yes = 1), Obstructive Sleep Apnea (No = 0, Yes = 1), Current Smoking or Smoked in the Past Status (No = 0, Yes = 1), Currently Working or Self-employed (No = 0, Yes = 1), Kids (No = 0, Yes = 1), Spouse (No = 0, Yes = 1), Living Alone (No = 0, Yes = 1), Daily Communication to Others (≤2 times =0, > 3 times = 1), Favorite Person to Talk: Friends (No = 0, Yes = 1), Weekly Walking (< 4 days = 0, ≥4 days = 1). Hosmer-Lemeshow test *p* = .642 (Total), *p* = .454 (YO), *p* = .986 (OO), the discriminant predictive value = 71.3% (Total), 63.7% (YO), 70.1% (OO), cut-off point: *GDS* Geriatric Depression Scale < 6 = 0, GDS ≥ 6 = 1, *p < 0.05, **p < 0.01. *CI* Confidence interval, *LL* Lower limit, *UL* Upper limit

## Discussion

The current study sought to examine factors related to the depression prevalence, and more specifically delineate the relationship between depression and social factors among older people living in the Siberian federal district of Russia. The results show that depression among YO adults is correlated with having asthma, having a spouse, and also less daily communication with others. Among OO adults, depression is correlated with not working and walking less than four times a week. This is the first study to show difference between social factors for depression prevalence, focusing on YO adults and OO adults separately in the Siberian federal district. As depression is an issue that spans many people worldwide, and especially many people in Russia, it is important to further qualify the characteristics of depression in different age groups to better target treatments.

### Depression prevalence among the older people in colder regions of Russia

Our results show 28.0% depression prevalence among study participants. The prevalence of depressive symptoms among older people in the European population ranges between 26 and 40% [18]. According to WHO (2017), 5.5% of the Russian population is diagnosed with depression [19]. In the relatively comparable study of the Russian population, with 16,877 participants, depression was found in 25.6% of the sample [13]. Depression quality and prevalence has been shown to change with age groups, and this study is the first of its kind to address the differences between age groups within a geriatric sample. In another study done in Russia, the inhabitants of Tomsk, another significant city in the Siberian federal district with even colder weather conditions than Novosibirisk, reported that 19.5% of participants showed ≥8 score on Hospital Anxiety and Depression Scale (HADS) [13]. In other high latitude countries like Russia, depression among older people aged 65 years and higher varies highly, from 9% in Norway and 12% in Sweden, to 14% in Canada and 16% in United Kingdom [20]. In colder regions, the higher risk of depression is often associated with fewer daylight hours per year [21] and low population density [22].

One point to note about depression prevalence overall is that it is known to be more common in woman than men, as shown through extensive former research. A previous report, which investigated the general population in 10 different regions across the Russian Federation, showed that depression prevalence in females was 28.6%, while in males it was 20.0% [13]. The results of the present study do not corroborate these statistics, as our study showed no significant difference in depression prevalence between genders (*p = 1.00*) in Novosibirsk oblast. A recent study by Akimova et al., under the leadership of World Health Organization, brought a new perspective on gender differences in depression prevalence in the colder regions of the Russian Federation. The epidemiological study’s age range involved groups between ages 25 and 64 in another cold region city, Tyumen, and showed that among all age groups depression was generally higher in females than in males [23]. Nevertheless, after the closer look at the older group (age 55–64), depression prevalence was statistically insignificant [23]. The authors discuss that social factors involved in the depression prevalence differs between age groups, thus mitigating statistical difference in depression prevalence between the genders in the older age groups, especially in the colder regions studied [23]. Our study also does not show significant difference between genders amongst older individuals, but sheds light on the more specific physical and social factors involved in depression prevalence within the older population of Novosibirsk oblast, a colder region in the Russian Federation.

### Main factors involved in depression among young old adults

The current study shows that frequent daily communication to others, i.e. more than 3 times a day, can drastically decrease the chances for depression in YO adults. Previous reports show that factors such as contact with family members and friends, change in human relationships, community involvement [24], social isolation and retirement are all social causes for depression in older age [25]. Among the older individuals in Russia, the social factors related to chronical illnesses play an especially large role in wellbeing, drastically changing communicative activity, lifestyle, and mental wellbeing, lessened contact outside the family or leading to exclusion from the social environment within the family [26]. Research on older individuals in another cold and low-density region in Finland showed that older people living alone or with somebody other than a partner were twice as likely to have depression than those living with a child and spouse [27]. Interestingly, our current results show that the existence of a spouse or kids was actually positively correlated with prevalence of depression only in YO adults. Based on pre-logistic analysis, the non-depressive group more frequently listed their favorite person with whom to talk as a friend, compared to others who listed spouse, kids, siblings, or other relatives (*P* = 0.009). In fact, living in low-density areas with little ability to interact with individuals outside the family strongly associated with depression, especially among older people [22]. Specific results concerning the family situation and its relation to one’s affective condition demonstrate potential regional differences, and therefore underscore the importance of the current research for future comparison and analysis.

### Main factors involved in depression among older old adults

For the OO adult participants in the study, depression was associated with different social factors. The results show that OO adults who walk more than 4 days a week are 3.5 times less likely to have depression. Previous studies also show the positive effects of daily walking, on both physical and mental health, reducing depression and improving quality of life [28, 29]. Even walking slightly less than 150 min per week can significantly contribute to reducing depression [30]. In fact, the previously mentioned lower depression prevalence (14%) among older individuals in Canada could be in part due to the fact that 68% of individuals aged 65 years and older walk outside more than 3 days per week [31]. Such prior research done concerning physical activity in relation to our study suggests that more physical activity is especially important in colder settings to lower depression risk or prevalence. However, physical activity among older people, especially in rural areas, significantly decreases in the colder seasons [32]. Despite the fact that decrease in activity of residents during the cold season in the study area covered in this study has not been investigated, it is without doubt that ecological factors of colder regions such as snow, ice, wind, and air quality play an important role in the ability to walk outside daily, especially among older people [33]. Beyond this form of physical activity, working or self-employment seems to play an even larger role in affecting depression for OO adults. Individuals who were currently working or self-employed had an 8.7 times lower odds ratio for depression. According to the Federal State Statistics Service, in the Russian Federation 23.1% of older people are employed after retirement or have paid work together with retirement income [34]. In 2018, a representative from the Ministry of Health of Russia stated that there is a 4.6 times higher risk of depression among unemployed older Russians [35], which is far lower than our results show. Novosibirsk oblast has approximately 1,360,200 individuals aged 55 and older who are employed; 74,400 60–69 year-olds and 11,300 people who are 70 and older [34]. In case of occupation, not only physical activity but also interaction with people and a sense of connection with society may affect depression. The importance of social ties differs with age, where marital status takes higher precedence for those aged under 60, whereas coworkers, close friends and/or relatives is considered more important for older individuals [36]. In addition, the aforementioned government representative stated that there is a 3 times increased risk for alcohol consumption among unemployed older people [35], significantly affecting depression prevalence within that group. According to a previous study of ours, among community-dwelling Russian older people, those who drank were 6 times more likely to talk about work than those who did not drink, demonstrating an active interest in the topic despite one’s age, and potentially using alcohol as a coping mechanism for not working [37]. Surprisingly, the current results demonstrate no significance in association between alcohol intake and depression (*P* = 0.628).

### Chronic illness and depression in cold regions

Various diseases and chronical illnesses could bring an excessive risk for depression [38]. In the current study, 4.5% of older people reported having asthma, and YO participants with asthma had 6.5 times higher relative odds for depression. Globally, the prevalence of self-reported asthma in older individuals is 4–13% [39]. Prevalence of asthma among general public Russians is relatively low at 2.6%, however the prevalence of asthma in the age group 60–69 reaches 7.6%, and the age group 70–79 has 9.9% asthma prevalence [40]. This is probably due to the fact that asthma severity is often considered to be age-related [41]. Previous studies show relatively higher risk (1.6 times) of depression among asthma patients compared to non-asthma participants [42], which is much lower than our results. That could be due to the fact that participants of the current study were living in a cold area, and asthma severity is especially affected by the climate of the cold and windy regions of Russia [43]. Previous research on the cold regions in Hokkaido, Japan, shows that more than 60% of patients with asthma in cold climates get worse during cold weather [9]. Moreover, other studies show that patients with asthma may perceive themselves as having more severe asthma than they actually have, thus they are more self-conscious of their health and depressive symptoms, therefore more often seek treatment [44, 45]. Although the current study did not investigate severity of asthma and self-consciousness regarding its symptoms, asthma patients are special target group among depressive older people, and controlling respiratory symptoms, especially in the cold climates, could potentially improve the quality of life and depression symptoms within that group [46, 47]. Nevertheless, an earlier study suggested no significant relationship between the severity of depression and the severity of asthma [48], and the causal relationship between asthma and depression is still unclear. Contradictive results on that topic and the low number of participants with asthma (*n* = 19) indicates that more data and research on this group is needed to arrive at a conclusion.

### Public policy and depression in colder regions of Russia

Lastly, monitoring depression in Russia as it relates to geographic and climatic factors is also an important consideration. In Russia, the mechanisms and services for screening depression varies unproportionally from oblast to oblast, and such services have not been reported to be used for diagnostical purposes [49]. Nevertheless, on the local basis in the Siberian Federal District, within the region of Tomsk oblast, there exists a unique program by Prof. Dr. Kornetov called “Recognition of Depression”, where psychiatrists and other doctors collaborate over screening of depression, anxiety, and suicide [50]. Probably because of this unique and local program, the level of suicide in Tomsk oblast decreased dramatically between 2008 and 2012 years by 54% [51]. In fact, Southern and Central Federal Districts show higher monitoring of depression on the regional basis, significantly impacting suicide prevention and depression diagnoses [2]. Distribution of social services based on regional knowledge and active depression monitoring from psychiatric services on the regional basis, are important tasks for current Russian governmental policies.

In Japan (a super-aging society), for comparison, exist several services geared towards screening of depression and suicide for older individuals. Such services exist at municipal health centers, health promotion centers, and welfare centers. The Visiting Nursing System (*homon kang*o) was developed under The Ministry of Health, Labour and Welfare of Japan, successfully advancing depression screening and increasing social interactions among older people [52]. Such services work not only for the geriatric population, but also for the caregiver network of these individuals, to prevent “burnout” of caregivers, which also influences the mental health of older people. Moreover, a telephone service for depressed individuals, called *kokoro no denwa,* consults callers and connects them to sufficient medical local and municipal facilities, maintaining anonymity and giving ability to obtain second opinions for diagnosis of mental well-being [53]. In fact, daily communication with others is already used as an effective treatment for depression, especially among older people [54], so telephone programs such as those in Japan can be a promising solution in the cold and low-density regions of Russia.

## Conclusion

Based on the findings present in this study, we conclude that depression among the older people in the colder regions of Russia is fairly common, albeit understudied. Frequent communication and social ties with individuals outside the family can mitigate depression prevalence, especially in YO adults. As for OO adults, the factors that have the highest impact on mitigating depression are related to daily activity, whether it be walking more frequently or working. Asthma patients are one of the more sensitive groups towards depression, but further research with larger sample sizes are needed to make conclusions about this target group. Social services and depression monitoring in cold and low-density regions are important tasks for Russian social policies, however increased sample size and future research on depression in Russia is required.

### Limitations

1. Only self-reported illnesses were used in this study, and the courses of treatment for the illnesses were unknown, whereas the current study did not assess the severity of asthma.
2. Since the presence of significant illness, cognitive impairment, and alcohol dependence can significantly affect depression prevalence among community-dwelling older people, those participants were excluded from the study.
3. Urban and rural differences of Siberian Federal District should be considered as a topic for future studies.
4. Biological factors of local and non-residential population are unclear and require future studies.
5. Due to no reports on the topic among the study region, current results compared the prevalence of depression in other provinces using different methods assessing depression.

## Supplementary Information

**Additional file 1.** [55]

## Data Availability

All data generated or analysed during this study are included in this published article [and its supplementary information files].

## References

[1] Statista (2016). Suicide mortality rate, leading countries worldwide.

[2] Astakhov P. Regions of Russia with high incidence of depression. RT. 2017; https://russian.rt.com/russia/news/456303-regiony-rossii-depressiya. Accessed 19 Apr 2020 (in Russian).

[3] Federal State Statistics Services of Russian Federation (2016). Population of the Siberian Federal District.

[4] Ministry of Natural Resources and Ecology of Russian Federation (2016). West-Siberian Federal State Service for hydrometeorology and environment monitoring.

[5] Lelkin MK, Selyatitskaya VG, Lutov YV (2009). Thyroid status disorders in working men and women depending on residence in the north. Bull SB RAMS.

[6] Hasnulin VI, Hasnulina AV (2012). Individual peculiarities of metabolic characteristics and resistance to psycho-emotional stress in the north. World Sci Cult Educ.

[7] Kot TL, Sirusina AV, Sirusina AV, Shalamova EU, Ragozin ON (2013). Latent factors of quality of life in patients with depressive disorders. Med Pharmaceut Mag "Pulse".

[8] Jogerst GJ (2006). Late life depression. Russian Fam Doctor.

[9] Osanai S, Takahashi T, Ogasa T, Nakano H, Ohsaki Y, Kikuchi K (2004). Symptoms in asthmatics living in cold districts during winter. Arerugi..

[10] Gottfries CG (1998). Is there a difference between elderly and younger patients with regard to the symptomatology and aetiology of depression?. Int Clin Psychopharmacol.

[11] Podkovina MI, Kostyuchkova ES, Bazhenova DS, Golovanova ED. Geriatric syndromes – prevalence, diagnosis, prevention: Vestnik of the Smolensk State Medical Academy; 2020. 10.37903/vsgma.2020:2.11.

[12] Oxenoit GK, Bougakova NS, Gelvanovski MI (2015). Russian regions. Social and economical indexes.

[13] Shal’nova SA, Evstifeeva SE, Deev AD, et al. The prevalence of anxiety and depression in different regions of the Russian Federation and its association with sociodemographic factors (according to the data of the ESSE-RF study). Ter Arkh. 2014. 10.17116/terarkh2014861253-60. 10.17116/terarkh2014861253-60 25804041

[14] Tombaugh TN, McIntyre NJ (1992). The mini-mental state examination: a comprehensive review. J Am Geriatr Soc.

[15] Ivanets NN, Kinkulkina MA, Avdeeva TI, Izyumina TA. Studying the possibilities of using standardized self-assessment scales of anxiety and depression in the examination of elderly patients: depression questionnaire scales. J Neurol Psych SS Korsakova. 2016. 10.17116/jnevro201611610151-59 (in Russian with English abstract).

[16] Backhaus J, Junghanns K, Broocks A, Riemann D, Hohagen F (2002). Test–retest reliability and validity of the Pittsburgh sleep quality index in primary insomnia. J Psychosom Res.

[17] Statista (2013). Mental quality of life among older people selected countries.

[18] Copeland R, Beekman AT, Braam AW (2003). Depression among older people in Europe: the EURODEP studies. World Psychiatry.

[19] World Health Organization (2017). Depression and other common mental disorders: global health estimates.

[20] Statista (2017). Seniors that experienced depression by country.

[21] Lam RW, Levit AJ (1999). Canadian consensus guidelines for the treatment of SAD. A summary of the report of the Canadian consensus group on SAD.

[22] Koohsari MJ, McCormack GR, Nakaya T (2019). Urban design and Japanese older adults’ depressive symptoms. Cities..

[23] Akimova EV, Akimov MJ, Gakova EI, Kayumova MM, Gafarov VV, Kuznetsov VA. Levels of depression and life exhaustion in the open population of the middle urbanized Siberian city: gender differences. Ter Arkh. 2019. 10.26442/00403660.2019.01.000028. 10.26442/00403660.2019.01.000028 31090371

[24] Katsumata Y, Arai A, Ishida K, Tomimori M, Lee RB, Tamashiro H (2012). Which categories of social and lifestyle activities moderate the association between negative life events and depressive symptoms among community-dwelling older adults in Japan?. Int Psychogeriatr.

[25] Grеbel’nik VI, Plyuhov AM (1997). On the diagnosis and hell of depression in the elderly. Soc Clin Psych.

[26] Shavlovskaya OA (2013). The medical social aspects of elderly age. Sociol Med.

[27] Joutsenniemi K, Martelin T, Martikainen P, Pirkola S, Koskinen S (2006). Living arrangements and mental health in Finland. Epidemiol Commun Health.

[28] Mobily KE, Rubenstein LM, Lemke JH, O’Hara MW, Wallace RB (1996). Walking and depression in a cohort of older adults: the Iowa 65+ rural health study. J Aging Phys Act.

[29] Lok N, Lok S, Canbaz M (2017). The effect of physical activity on depressive symptoms and quality of life among elderly nursing home residents: randomized controlled trial. Arch Gerontol Geriatr.

[30] Mammen G, Faulkner G (2013). Physical activity and the prevention of depression: a systematic review of prospective studies. Am J Prev Med.

[31] Government of Canada (2009). Walking data, from Canadian community health survey.

[32] Nakashima D, Kimura D, Watanabe H (2019). Influence of seasonal variations on physical activity in older people living in mountainous agricultural areas. J Rural Med.

[33] King AC, Sallis JF, Frank LD (2011). Aging in neighborhoods differing in walkability and income: associations with physical activity and obesity in older adults. Soc Sci Med.

[34] Federal State Statistics Services of Russian Federation (2019). Population.

[35] Tkacheva O (2018). Life expectancy in Russia will exceed 80 years by 2030.

[36] Seeman TE, Kaplan GA, Knudsen L, Cohen R, Guralnik J (1987). Social network ties and mortality among the elderly in the Alameda County study. Am J Epidemiol.

[37] von Fingerhut G, Matsuda H, Okamoto N (2019). Influence of drinking on sleep, physical health and social interactions in the Russian community dwelling elderly in urban areas. Pacific Med J.

[38] Park JI, Park TW, Yang JC, Chung SK (2016). Factors associated with depression among elderly Koreans: the role of chronic illness, subjective health status, and cognitive impairment. Psychogeriatrics..

[39] Dunn RM, Busse PJ, Wechsler ME (2018). Asthma in the elderly and late-onset adult asthma. Allergy..

[40] Maximova T, Belov V, Lushkina N, Karpova V (2014). Study on global ageing and adult health (SAGE) wave 1, Russian Federation National Report.

[41] Morris LTCMJ (1999). Difficulties with diagnosing asthma in the elderly. Chest.

[42] Scott KM, von Korff M, Ormel J, Zhang M, Bruffaerts R, Alons J (2007). Mental disorders among adults with asthma: results from the world mental health survey. Gen Hosp Psychiatry.

[43] Chuchalin AG (2001). Severe forms of bronchial asthma. Therapeutic Arch.

[44] Zielinski T, Brown S, Nejtek V, Kahn D, Moore J, Rush A (2000). Depression in asthma: prevalence and clinical implications. Prim Care Compan J Clin Psych.

[45] Walters P, Schofield P, Howard L, Ashworth M, Tylee A (2011). The relationship between asthma and depression in primary care patients: a historical cohort and nested case control study. PLoS One.

[46] Ford ES, Mannino DM, Homa DM, Gwynn C, Redd SC, Moriarty DG (2003). Self-reported asthma and health-related quality of life: findings from the behavioral risk factor surveillance system. Chest..

[47] Voros V, Martin Gutierrez D, Alvarez F, Boda-Jorg A, Kovacs A, Tenyi T (2019). The impact of depressive mood and cognitive impairment on quality of life of the elderly. Psychogeriatrics..

[48] Janson C, Bjornsson E, Hetta J, Boman G (1994). Anxiety and depression in relation to respiratory symptoms and asthma. Am J Respir Crit Care Med.

[49] Budyakova TP, Pronina AN, Baturkina GV (2019). The role of physical culture and sports in ensuring the life-resistance of elderly and persons of pre-pension age. Sci Notes Univ PF Lesgaft.

[50] Kornetov NA (2013). A multi-aspect model of suicide prevention. Tyumen Med J.

[51] Lyubov EB, Kabizulov VS, Tsuprun VE, Chubina SA (2014). Territorial suicidological services of the Russian Federation: structure and function. Suicidology..

[52] Japan Visiting Nursing Foundation (2015). Visiting nursing system in Japan.

[53] Ministry of Health, Labour and Welfare of Japan (2009). About depression of elderly people.

[54] Carey B (2006). Talk therapists cite major strides in fending off depression among the elderly.

[55] Ware JE, Kosinski M, Dewey JE, Gandek B (2001). How to score and interpret single-item health status measures: a manual for users of the SF-8 health survey.

