# Supplementary material for: Social and physical factors related to depression in the older population of Siberia, Russia: a cross-sectional study
Source: BMC Geriatr. 2021 Apr 23;21:272. doi: 10.1186/s12877-021-02225-7 (PMC8066855; doi:10.1186/s12877-021-02225-7)
Supplement: Supplementary file 1 — Additional file 1. [55] [file 12877_2021_2225_MOESM1_ESM.docx]

**SUPPLEMENTARY FILE 1**

**[Title Page]**

**Title:** Social and Physical Factors Related to Depression in the Older Population of Siberia, Russia: A Cross-Sectional Study

**Authors:** Georg von Fingerhut*^1^, Katsuyoshi Mizukami^2^, Dorothy Yam^3^, Konstantin Makarov^4^, Yuriy Kim^5^, Elena Kondyurina^6^, Lyudmila Yakovleva^7^.

**1**. How old are you?　　　　　years old

**2**. Gender：0．Male　1．Female

**3**. Hight：　　　　　　㎝

**4**. Weight：　　　　　　㎏

**5.** Do you have anything from the following diseases (multiple answer):

1. Osteosclerosis, 2. Diabetes, 3. Asthma, 4. Kidney Disease, 5. Digestion Disorder, 6. Hypertonia, 7. Cardio-Vascular Diseases, 8. Obstructive Sleep Apnea, 9. Other _________

**6**．Currently, do you have a spouse? 1. Yes 2. No

**7**. Currently, do you have kids? 1. Yes 2. No

**9**. Who do you currently live with? 1. Living alone 2. Living with somebody

**10.** Please indicate years of completed education: ____ years

**11.** Please indicate the level of your monthly income: 1. Lower than 8,524 rub. 2. Higher than 8,524 rub

**12**. Please indicate your occupation: 1. Unemployed 2. Currently Working or Self-employed

**13.** Do you currently smoke cigarettes or have you ever smoked before? 1. I am currently smoking 2. I have smoked in the past. 3. I do not smoke

**14.** Do you drink alcohol? 1. Yes 2. No

**15.** Please indicate how often do you communicate with other people during one day? ____ times

**16.** Please indicate the amount of time you spend communicating at one time: _____ minutes

**17.** Who is the most pleasant communication partner for you now? (one answer) 1. Spouse 2. Kids 3. Siblings 4. Relatives 5. Friends 6. Other _________

**18.** How often do you go out for a walk per week? ___ days per week

**19.** The 15-items Geriatric Depression Scale ^10^

**20.** The Pittsburgh Sleep Quality Index ^11^

**21.** Do you ever take a nap? * nap is a dream of your own free will during the day. If yes, how long do you nap or sleep during the day in general _____ minutes

**22.** The 8-item Short-Form Health Survey ^55^
